# Gastrointestinal Tuberculosis Presenting as Malnutrition and Distal Colonic Bowel Obstruction

**DOI:** 10.1155/2018/2808565

**Published:** 2018-02-27

**Authors:** Raja Chandra Chakinala, Zahava C. Farkas, Benjamin Barbash, Khwaja F. Haq, Shantanu Solanki, Muhammad Ali Khan, Edward Esses, Taliya Farooq, Brad Dworkin

**Affiliations:** ^1^Medicine, New York Medical College, Westchester Medical Center, Valhalla, NY, USA; ^2^Division of Gastroenterology and Hepatobiliary Diseases, New York Medical College, Westchester Medical Center, Valhalla, NY, USA; ^3^Division of Gastroenterology, University of Tennessee Health Science Center, Memphis, TN, USA; ^4^Department of Radiology, New York Medical College, Westchester Medical Center, Valhalla, NY, USA; ^5^Department of Pathology, New York Medical College, Westchester Medical Center, Valhalla, NY, USA

## Abstract

Gastrointestinal (GI) tuberculosis (TB) is rare and can occur in the context of active pulmonary disease or as a primary infection with no pulmonary symptoms. It typically presents with vague abdominal symptoms, making it difficult to discern from alternative disease processes. Although the ileocecal region is the most commonly affected site, tuberculous enteritis can involve any aspect of the GI tract. To demonstrate the importance of maintaining a high clinical suspicion for the disease, we present a case of GI TB presenting as severe malnutrition and segmental colitis of the left colon.

## 1. Introduction

Extrapulmonary TB occurs in approximately 20% of TB cases in immunocompetent patients, with tuberculous enteritis accounting for approximately 1–3% of TB cases worldwide. The bacterium is thought to enter the GI tract via hematogenous spread from active pulmonary TB, from swallowing infected sputum or from ingestion of contaminated food products [1–4]. The ileocecal region is the most commonly affected site; however, any portion of the GI tract can be involved. Penetration of the GI mucosa leads to an inflammatory reaction with subsequent granuloma formation, mucosal ulceration, and necrosis. As the signs and symptoms of tuberculous enteritis are typically vague, the diagnosis is often difficult to make and requires a high index of suspicion [2, 3].

## 2. Case Report

**Figure 1:**
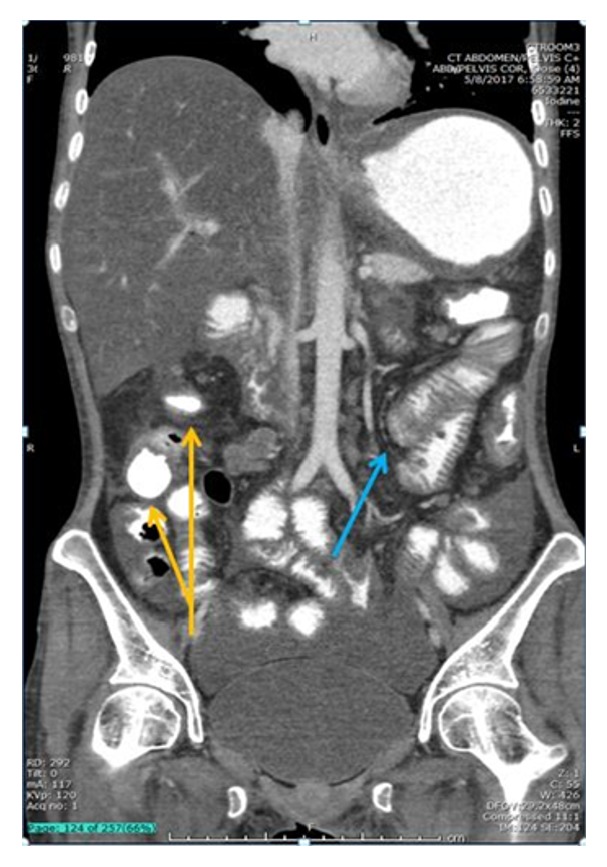
CT demonstrating concentric bowel wall thickening in the region of the splenic flexure (blue arrow) with sparing of the region of the hepatic flexure (yellow arrow). The patient later developed colonic obstruction at both sites.

**Figure 2:**
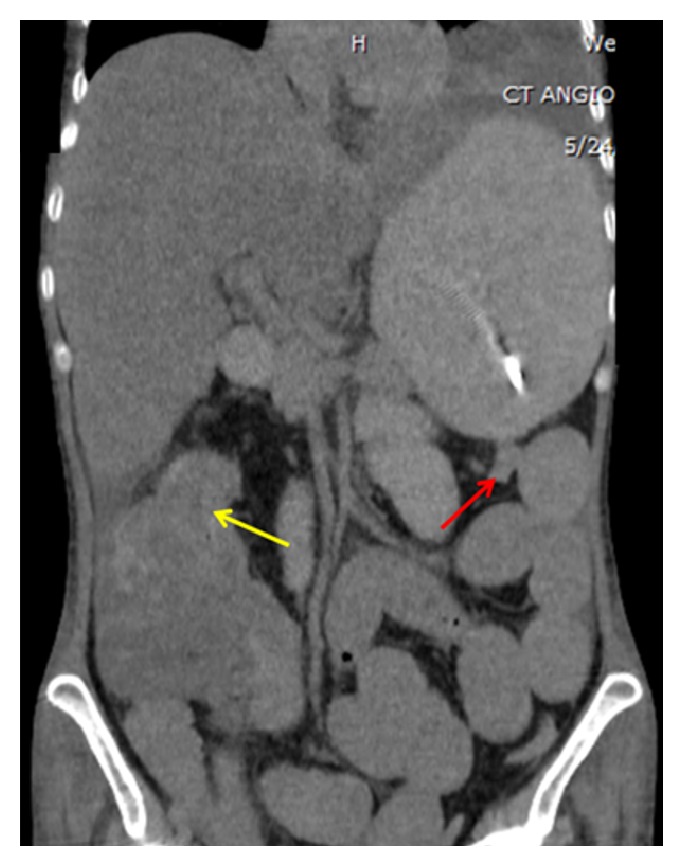
CT demonstrating distal luminal narrowing of bowel loops at both the splenic flexure (red arrow) and hepatic flexure (yellow arrow) with associated bowel wall thickening and edema.

**Figure 3:**
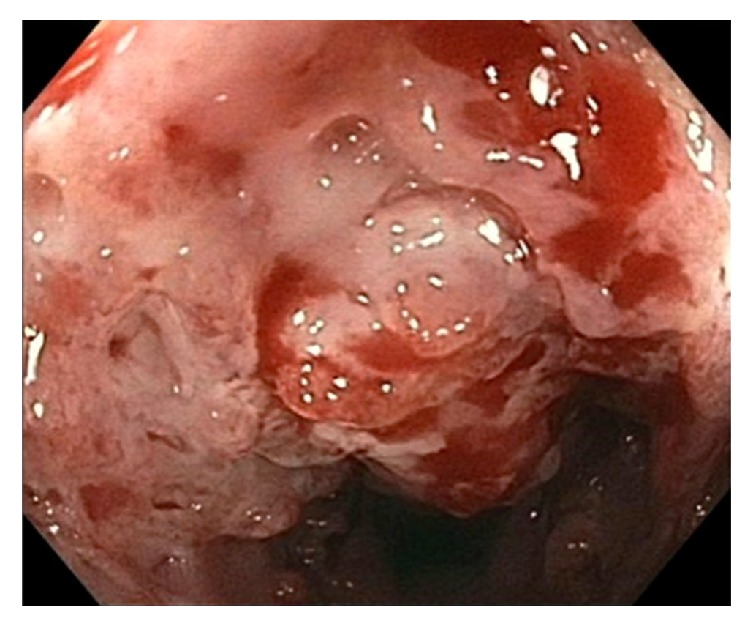
Colonoscopy image showing circumferential friable ulcerated mucosa in the descending colon.

**Figure 4:**
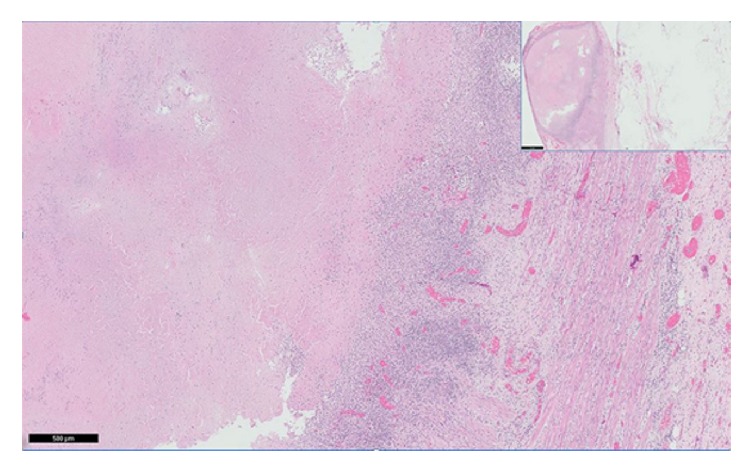
Histopathology of full thickness section of colon with pericolonic fat showing confluent necrotizing granulomatous inflammation involving transmural colonic wall.

**Figure 5:**
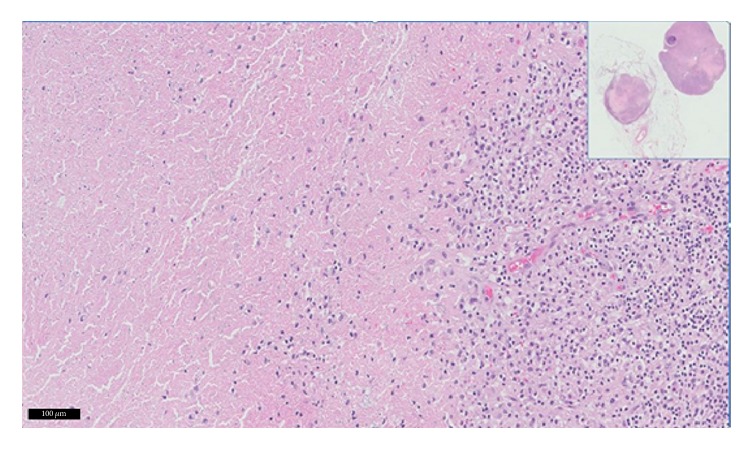
2 lymph nodes with confluent necrotizing granulomatous inflammation as visualized at 1x (inset) and 20x.

A 36-year-old woman who emigrated from the Ecuador in 2002 presented to the emergency department with a one-year history of intermittent abdominal pain, diarrhea, and a 70-pound weight loss. She had no reported medical history, recent travel outside of the US, or known sick contacts. She appeared cachectic with diffuse muscle wasting and had a body mass index (BMI) of 14. Initial labs were notable for a white blood cell count 4.5 k/mm^3^, hemoglobin 8.8 g/dL, iron 17 mcg/dL, TIBC 47 mcg/dL, ferritin 123  *μ*g/L, C-reactive protein 9.4 mg/dL, HIV negative, anti-nuclear antibody screen negative, anti-myeloperoxidase antibody negative, anti-perinuclear-3-antibody negative, and albumin 1.8 g/dL. Computed tomography (CT) of the chest and abdomen showed several right-sided pulmonary nodules, a large left-sided pleural effusion, and diffuse small and large bowel wall thickening with enlarged mesenteric lymph nodes. EGD revealed atrophic mucosa throughout the stomach and proximal duodenum. Esophageal, gastric, and duodenal biopsies were unrevealing. Colonoscopy showed circumferential friable ulcerated mucosa in the descending colon (Figure 3). The colonoscope was not advanced beyond this area. Biopsies showed necrotic tissue that was negative for acid-fast bacilli (AFB). Extensive autoimmune and infectious workup was unrevealing.* Mycobacterium tuberculosis* (MTB) polymerase chain reaction from an induced sputum sample was positive. Stool culture was positive for acid-fast bacilli. Soon after antituberculin therapy was initiated she developed a large bowel obstruction with segmental colitis and strictures at multiple areas including the splenic and hepatic flexures as seen on CT scan (Figures 1 and 2), and she required a subtotal colectomy with end-ileostomy. Pathology of the resected colon showed confluent necrotizing granulomatous inflammation with transmural colonic wall involvement and stricture at the splenic flexure, as well as necrotizing granulomas of the surrounding lymph nodes, consistent with tuberculosis (Figures 4 and 5). There was no inflammation or pathology in the terminal ileum. She was continued on antituberculin therapy and was discharged to a rehabilitation facility with improvement in her symptoms and malnutrition.

## 3. Discussion

TB enteritis typically presents with vague clinical, radiographic, and histopathologic findings that mimic malignancy and inflammatory bowel disease [5]. Maintaining a high index of suspicion is key as the diagnosis is difficult to establish and one-year mortality rates are as high as 20 percent [2, 3, 6]. Diagnosing exclusive intestinal TB is difficult and often made by combining clinical suspicion, stool and tissue AFB staining, and tissue histology. A presumptive diagnosis of TB enteritis can also be made in the setting of active pulmonary TB together with clinical, endoscopic, and/or radiographic findings of intestinal TB.

Chronic abdominal pain is the most common symptom along with anorexia, fatigue, night sweats, diarrhea, or blood in the stool. Routine lab work is usually nonspecific showing mild anemia and elevated inflammatory markers. Mycobacterial cultures of the sputum or stool yield positive results approximately 50% of the time. In cases of intestinal TB, AFB and MTB culture positivity on intestinal biopsies is rarely found, in only 17% and 29% of cases, respectively, in one large study [7].

The most common radiographic finding is concentric mural thickening of the ileocecal region with or without proximal intestinal dilatation [5, 8, 9]. The ileocecal region accounts for almost 64% of cases of GI TB. The affinity of MTB for this site may be due to relative stasis and abundant lymphoid tissue in this area. As in the above reported case, bowel obstruction is the most common complication. Our patient had segmental colitis with involvement of the splenic flexure, an atypical presentation of GI TB.

Circumferential ulcers surrounded by inflamed mucosa are common colonoscopic findings in GI TB. Classically reported histopathologic findings include large, confluent granulomas with caseating necrosis; however, caseating granulomas and acid-fast bacilli are found in less than 33% of cases. In a significant number of cases (44.5%), granulomas were seen in a submucosal location, and the predominant type of inflammation seen in the lamina propria was lymphoplasmacytic [1]. Antimicrobial therapy (RIPE: rifampicin, isoniazid, pyrazinamide, and ethambutol for two months followed by rifampicin plus isoniazid for an additional six months) remains the mainstay of the treatment for GI TB; however, surgical or endoscopic intervention is often required in cases complicated by perforation or obstruction [10]. In one study, colonoscopic follow-up after 2-3 months of anti-TB therapy showed complete healing of the ulcers and erosions [11, 12].

Our immunocompetent patient with disseminated TB presenting with exclusive GI symptoms was found to have left-sided and later right-sided colon involvement leading to bowel obstruction. This is an atypical manifestation of tuberculosis with a rare clinical presentation. It is essential to maintain a broad differential diagnosis when evaluating a patient with vague symptoms and nonspecific test results, while paying close attention to patient demographics and risk factors.
